# Effects of fish oil and spirulina on oxidative stress and inflammation in hypercholesterolemic hamsters

**DOI:** 10.1186/1472-6882-14-470

**Published:** 2014-12-06

**Authors:** Miriam Adoyo Muga, Jane C-J Chao

**Affiliations:** School of Nutrition and Health Sciences, College of Public Health and Nutrition, Taipei Medical University, 250 Wu-Hsing Street, Taipei, 110 Taiwan; Master Program in Global Health and Development, College of Public Health and Nutrition, Taipei Medical University, Taipei, 110 Taiwan; Nutrition Research Center, Taipei Medical University Hospital, Taipei, 110 Taiwan

**Keywords:** Hypercholesterolemia, Spirulina, Fish oil, Oxidative stress, Inflammation

## Abstract

**Background:**

Altered plasma lipids, oxidative stress, and inflammation have been involved in the pathogenesis of cardiovascular disease. Fish oil has shown inconclusive effects on plasma lipids and oxidative stress. Spirulina has both cholesterol lowering and antioxidant properties. However, the effect of fish oil and spirulina on hypercholesterolemia has not been studied. We investigated the effects of fish oil, spirulina, and their combination on hypercholesterolemia.

**Methods:**

The hamsters were divided into 7 groups: control, high cholesterol (HF), fish oil (post FO), spirulina (post SP), and a combination of fish oil and spirulina (post SF, pre-SF, and HF + SF) groups. The HF and HF + SF groups were given a high cholesterol diet for 8 weeks. The post FO, post SP, and post SF groups were given a high cholesterol diet for 4 weeks and then the treatment for 4 weeks. The pre-SF group was given the combined treatment for 4 weeks and then a high cholesterol diet for 4 weeks.

**Results:**

The HF and HF + SF groups altered plasma lipids, increased oxidative stress, inhibited antioxidants, and increased inflammation. While the post FO group increased plasma lipids and was more atherogenic. The vice versa was observed in spirulina-treated group. Both the post SP and post SF groups inhibited oxidative stress and increased antioxidant status, and post FO and post SP diets regulated pro-inflammatory cytokines to near the control levels.

**Conclusions:**

Both single treatment of fish oil or spirulina inhibit oxidative stress and inflammation. Treatment with a combination of fish oil and spirulina (post SF) may be beneficial for diet-induced hypercholesterolemic hamsters.

## Background

Cardiovascular disease (CVD) is the most common cause of mortality and morbidity in the West, accounting for more than 50% of all deaths [1]. Hypercholesterolemia on the other hand has been identified as a critical step in the development of atherosclerosis and a predisposing risk factor for CVD [2]. Various studies have shown a positive correlation between cholesterol levels and the risk of hypercholesterolemia and hence characterizing it by elevated levels of LDL-C and total cholesterol and low level of HDL-C [2–6]. Furthermore, the elevated cholesterol levels sets stage for the formation of free oxygen radicals hence increasing oxidative stress and suppressing the antioxidant enzymes. Both clinical and animal studies have shown increased lipid peroxidation in plasma and a poor antioxidant defense system in the presence of hypercholesterolemia [7–9].

Some of the dietary management methods for CVD include the use of fish oil and positive effects in human studies and in other animal models have been observed [10–15]. However, in hamsters it has shown opposite results by increasing their lipid profiles in the presence of a high cholesterol diet; an effect that is attributed to the decrease in hepatic LDL-receptor mRNA expression [16–18]. Furthermore, both animal and human studies show conflicting results on the ability of fish oil to prevent formation of free radicals and to improve the antioxidant status of subjects [19–24]. Therefore, it is still unclear whether fish oil with its high peroxidation ability can still lower blood lipids and exhibit antioxidant properties. Nevertheless, in-vitro studies have shown that fish oil can regulate inflammation by inhibiting the production of pro-inflammatory cytokines and adhesion molecules related to atherosclerosis [25, 26].

On the other hand, antioxidant vitamin is beneficial for cardiovascular patients because of their ability to inhibit oxidation or the uptake of LDL cholesterol [27]. However, the effects of antioxidants on cardiovascular health have shown conflicting results [28]. The most common form of antioxidant used with fish oil is vitamin E because of its anti-atherogenic ability to inhibit LDL oxidation [9, 29, 30]. However, the evidence suggests that fish oil greatly lowers plasma vitamin E level in hypercholesterolemic subjects. The recent animal study observed the effect of stress on the antioxidant status of animals and found that stress increased the level of lipid peroxides and reduced the levels of antioxidants (vitamin E and L-ascorbic acid) in liver and kidney [31]. The previous studies showed that fish oil combined with vitamin E significantly reduced the concentration of vitamin E in the body [32, 33]. However, few studies have demonstrated the effects of water-soluble antioxidants combined with fish oil on hypercholesterolemia. Spirulina, a green-blue algae, was used in this study, because it had cholesterol-lowering ability, protected against free radicals and cell death, and increased antioxidant enzyme activities in both plasma and liver [34–37]. However, the effects of spirulina on inflammatory cytokines in hypercholesterolimic and atherogenic conditions have not been elucidated.

The objective of this study was to compare the effects of single treatment of fish oil or spirulina and their combination on plasma lipids, oxidative stress, and inflammation before and after the induction of hypercholesterolemia in hamsters.

## Methods

### Experimental hamsters

Male Golden Syrian hamsters (n = 84) were obtained from National laboratory Animal Center (Taiwan). The hamsters were 8-week old at the time of their delivery. The animals were fed on a commercial rodent chow diet for 3 days to be acclimated to the facility before the commencement of the experiment. Hamsters were housed in stainless steel and wired-bottomed hanging cages in an environmentally controlled atmosphere (23°C) on a 12-h light:dark cycle. Hamsters consumed their feed and water *ad libitum* before and during the experimental period. All animal protocols were approved and in accordance with the guidelines of the Institutional Animal Care and Use Committee of Taipei Medical University.

### Diets and treatments

On the third day after delivery of the hamsters to the institution, their diet was switched from the standard rodent chow to AIN-93 M diet for 7 days. The AIN-93 M diet has a balance of essential nutrients and is suitable for both long- and short-term studies with laboratory rodents. While the control group (C) was given the AIN-93 M diet, all other experimental diets were modified from the basic AIN-93 M diet. The high cholesterol diet (HF) was prepared by adding 7.5% cocoa butter and 1.25% cholesterol to the AIN-93 M diet (Table 1) [38]. In addition, 0.08 g/kg feed of fish oil from menhaden fish (Sigma-Aldrich China, Inc., Shanghai, China) and 0.011 g/kg feed of spirulina (Fuqing King Dnarmsa Spirulina Co., Ltd, FuQing, China) were subsequently added by small amounts with continuous stirring to the AIN-93 M diet to constitute the fish oil (FO) and spirulina (SP) diets, respectively. The same doses of fish oil and spirulina were added together to either the AIN-93 M or to the HF diet to form the diets for combined treatments (SF and HF + SF). Fatty acid compositions in menhaden fish oil (Sigma-Aldrich China) are composed of approximately 80% identified fatty acids, including 6-9% myristic acid (14:0), 15-20% palmitic acid (16:0), 9-14% palmitoleic acid (16:1), 3-4% stearic acid (18:0), 5-12% oleic acid (18:1), < 3% linoleic acid (18:2), < 3% linolenic acid (18:3), 2-4% octadecatetraenoic acid (18:4), < 3% arachidonic acid (20:4), 10-15% eicosapentaenoic acid (EPA, 20:5), and 8-15% docosahexaenoic acid (DHA, 22:6), and 20% unidentified fatty acids. Spirulina powder (Fuqing King Dnarmsa Spirulina Co., Ltd) primarily contains 65.2% protein, 6.6% water, 6.7% ash, 13.7% crude ohycocyanin, 1241 mg/100 g chlorophyll, and 463 mg/100 g total carotenoids.

**Table 1:** **Compositions of the standard and hypercholesterolemic diets**

Ingredient (g/kg feed)	Standard AIN-93 M diet	Hypercholesterolemic diet
Casein	141.40	141.40
Corn starch	470.35	470.35
Alphacel	50.50	50.50
Soybean oil	40.40	40.40
L-cystine	1.82	1.82
Choline bitartrate	2.53	2.53
Tert-butylhydroquinone	0.0081	0.0081
Sucrose	101.00	101.00
Dextrinized cornstarch	156.55	156.55
Mineral/vitamin mix	35.45	35.45
Cholesterol	0.00	0.0126
Cocoa butter	0.00	0.076

After the acclimatization period, hamsters were randomly divided into 7 groups and housed in groups of 4 per cage. Hamsters in each group except the control group received a high cholesterol diet with either fish oil, spirulina, or a combination of fish oil and spirulina as follows: control (C, n = 12), high cholesterol (HF, n = 12), fish oil (post FO, n = 12), spirulina (post SP, n = 12), spirulina and fish oil (post SF, n = 12), treatment then the induction of hypercholesterolemia (pre-SF, n = 12), and the induction of hypercholesterolemia + treatment (HF + SF, n = 12) groups. The post FO, post SP, and post SF groups were first fed a high cholesterol diet to induce hypercholesterolemia for 4 weeks followed by different treatments for the remaining 4 weeks. The pre-SF group was first fed a combination of spirulina and fish oil for 4 weeks and then a high cholesterol diet for the last 4 weeks. The comparisons between the high cholesterol diet and control groups were to determine whether the induction of hypercholesterolemia was successful by primarily measuring plasma lipid levels. Additionally, since the hamsters had different treatments with different feeding periods, all treatment groups were compared with the control group which served as the baseline for the values of the dependent variables to determine whether the treatments had significant effects compared with the control hamsters with normal dependent variables. The HF + SF group was compared with the HF group because both groups were given a high cholesterol diet for 8 weeks with or without treatment, respectively, and the post FO, post SP, post SF, and pre-SF groups were compared with each other to study the pre- and post-treatment effects of fish oil and/or spirulina. Body weight of the hamsters was recorded weekly and at the 8th week of the study period. Blood, liver, and aorta samples were collected from food-deprived (16 h) hamsters for plasma lipids, aortic cholesterol concentration, TNF-α, IL-1β, and IL-10 in the aorta, antioxidants (total glutathione, GSH; glutathione peroxidase, GSHPx; superoxide dismutase, SOD), and lipid peroxidation (thiobarbituric acid reactive substances, TBARS) in liver.

### Measurements of plasma lipids

Blood was drawn in heparinized tubes by heart punctuation and placed on ice. The collected blood was then centrifuged at 1500× *g* at 4°C for 10 min. Plasma supernatant was used for the determination of total cholesterol (TC), triglycerides (TG), and high density lipoprotein-cholesterol (HDL-C) using enzymatic kits purchased from Randox Laboratories (Antrim, Northern Ireland, UK). Low density lipoprotein-cholesterol (LDL-C) was calculated as the difference between plasma TC and HDL-C. The ratio of plasma TC to HDL-C was then calculated, and the atherogenic index of plasma (AIP) was determined as Log (TG/HDL-C) for the risk of cardiovascular disease [39].

### Aortic cholesterol levels

At the end of the exposure period (week 8), hamsters were anesthetized with intraperitoneal injection of sodium pento-barbital, and aortic tissue was obtained for the determination of cholesterol and cytokine concentrations. A section of aortic tissue (20–40 mg) extended from as close to the heart as possible to the branch of the left subclavian artery was dissected, minced, and homogenized in phosphate-buffered saline (pH 7.0). The homogenized tissue was centrifuged at 400× *g* for 10 min. The supernatant was collected and stored at −20°C.

Aortic lipids were extracted according to the modified method of Alexaki et al. [40]. Methanol (4 ml) and chloroform (8 ml) were separately added to aortic homogenate. The solution (3 ml) containing 1.25% KCl and 0.05% H_2_SO_4_ was then added and centrifuged at 400× *g* at room temperature for 10 min. The bottom layer was transferred and the supernatant was re-extracted with 3 ml of chloroform:methanol (2:1) and centrifuged at 400× *g* at room temperature for 10 min. The bottom layer was transferred and pooled with that from the previous step. The chloroform solution was stored at −20°C until analysis.

After chloroform was completely evaporated, chloroform (1 ml) with 1% Triton-100 was added, mixed, and completely evaporated. Distilled water (500 μl) was then added, and the sample was placed in a shaking water bath at 37°C for 20 min to solubilize lipids. After incubation, aortic total cholesterol concentration was determined enzymatically using commercial kits (Randox Laboratories).

### Hepatic lipid peroxidation

Liver sample (0.3 g) was homogenized in 1.2 ml of 50 mM Tris–HCl. The homogenate was then centrifuged at 8500× g at 4°C for 10 min, and the supernatant was used for biochemical analysis. Protein concentration in each fraction was determined by the method of Lowry et al. [41]. The mean thiobarbituric acid reactive substances (TBARS) (μmol/mg protein), a measure of lipid peroxidation, was assayed in the form of malondialdehyde (MDA) using a commercial kit purchased from Cayman Chemical Company (Ann Arbor, MI, USA).

### Liver glutathione (GSH) level and glutathione peroxidase (GSHPx) activity

Liver samples (0.3 g) were homogenized in 5% metaphosphoric acid (pH 7.0). The homogenate was then centrifuged at 3000× *g* for 10 min, and the supernatant was used for biochemical analysis. Protein concentration in each fraction was determined by the method of Lowry et al. [41]. Glutathione level was assayed using a commercial kit purchased from Cayman Chemical Company, and GSHPx activity was determined using a commercial kit purchased from Randox Laboratories.

### Liver superoxide dismutase (SOD) activity

Liver samples (0.3 g) for the determination of SOD activity were homogenized in a solution containing 0.25 M sucrose, 50 mM Tris HCl, and 5 mM EDTA (pH 7.0). The homogenate was then centrifuged at 8500× *g* at 4°C for 10 min, and the supernatant was used for biochemical analysis. Liver SOD activity was expressed as activity per mg protein. Protein concentration in each fraction was determined by the method of Lowry et al. [41]. The activity of superoxide dismutase was assayed using a commercial kit purchased from Randox Laboratories.

### Aortic TNF-α, IL-1β, and IL-10 levels

The proinflammatory (TNF-α and IL-1β) and anti-inflammatory (IL-10) cytokines were measured using commercial kits (Shangai BlueGene Biotech Co., Shanghai, China). Standards or samples (50 μl) were added in the antibody pre-coated plate followed by 100 μl of conjugate solution and incubated for 1 h at 37°C. Reactive substrates were added for 10 min at 20-25°C, and the optical density was read at 450 nm using an ELISA microplate reader.

### Statistical analysis

The data are expressed as mean ± SD. One-way analysis of variance (ANOVA) and Fisher’s least significant difference test were used for the differences in means using Statistical Analysis System (SAS version 9.3, SAS Institute Inc., Cary, NC, USA). *P*-value < 0.05 was considered statistically significant.

## Results

### General characteristics of the hamsters

Body weights were not significantly different at week 0 among different groups, and not significantly different among the control, HF, post FO, post SP, and post SF groups throughout the experimental period (Table 2). While the HF+ SF group had lower body weights compared with the control group throughout the experimental period (*p* < 0.05), the pre-SF group had higher body weights compared with all post-treatment groups at week 4 (*p* < 0.05). However, despite the positive increase in body weight in all groups at week 8, the HF + SF group still had significantly lower body weight and weight gain compared with the control group (*p* < 0.05). Average food intake was decreased in the post FO, post SF, pre-SF, and HF + SF groups compared with the control and HF groups (*p* < 0.05). Reduced food intake in all fish oil groups may be attributed to the increased satiety by fat content in fish oil diet and the strong flavor of fish oil. The reduction in food intake may partially explain lower body weight and weight gain in the HF + SF group. While food efficiency in the HF and HF + SF groups was lower compared with the control group (*p* < 0.05), and that in the post SF and pre- SF groups was lower compared with the control and post SP groups. The post SP group restored food efficiency to the similar level as the control group. The induction of hypercholesterolemia at the beginning of the experiment had a significant effect on liver weight. All experimental groups had an increase in liver wet weight when compared with the control group (*p* < 0.05). However, the post FO, post SP, and post SF groups had significantly lower liver weights compared with the pre-SF group (*p* < 0.05). On the other hand, the relative liver weights of all experimental groups were significantly higher than the control group, and the post SP group had significantly lower relative liver weights than the post SF and pre-SF groups (*p* < 0.05). Aorta wet weight was not different in all experimental groups compared with the control group. While the HF, HF + SF, post FO, and post SF groups had increased relative aorta weight compared with the control group (*p* < 0.05), there were no differences in relative aortic weight among the post SP, pre-SF, and the control groups (*p* < 0.05).

**Table 2:** **Body, liver, and aorta weights of hamsters in different groups**

	Control	HF	Post FO	Post SP	Post SF	Pre-SF	HF + SF
Body weight, g							
Week 0	110.2 ± 3.3^a^	112.6 ± 8.5^a^	112.0 ± 12.3^a^	111.7 ± 9.5^a^	112.2 ± 9.3^a^	112.6 ± 7.8^a^	110.5 ± 11.1^a^
Week 4	120.1 ± 11.0^b^	113.9 ± 10.0^ab^	112.3 ± 11.6^ab^	114.2 ± 11.7^ab^	113.5 ± 12.6^ab^	129.7 ± 10.0^c^	110.1 ± 8.6^a^
Week 8	134.3 ± 14.6^b^	117.4 ± 10.5^ab^	119.0 ± 12.6^ab^	125.2 ± 8.9^b^	118.1 ± 12.0^ab^	132.5 ± 10.1^b^	110.9 ± 8.8^a^
Change in body weight, g	14.2 ± 5.7^c^	3.5 ± 8.5^ab^	6.7 ± 4.2^b^	10.9 ± 7.2^bc^	4.7 ± 6.9^ab^	2.9 ± 6.7^ab^	0.8 ± 2.8^a^
Food intake, g/day	7.6 ± 0.1^d^	7.5 ± 0.3^d^	6.9 ± 0.1^b^	7.5 ± 0.1^d^	6.6 ± 0.3^b^	7.2 ± 0.2^c^	5.8 ± 0.6^a^
Food efficiency, %	7.6 ± 3.3^c^	0.5 ± 1.3^ab^	3.6 ± 2.3^bc^	5.4 ± 3.6^c^	2.6 ± 3.9^b^	1.5 ± 3.4^ab^	0.5 ± 1.9^a^
Liver wet weight, g	4.3 ± 0.7^a^	7.3 ± 0.8^c^	6.1 ± 0.7^b^	6.0 ± 0.9^b^	6.2 ± 0.8^b^	7.1 ± 0.8^c^	6.8 ± 0.6^c^
Relative liver weight, %	3.2 ± 0.3^a^	6.4 ± 0.7^d^	5.1 ± 0.3^bc^	4.8 ± 0.5^b^	5.3 ± 0.5^c^	5.4 ± 0.3^c^	6.1 ± 0.5^d^
Aorta wet weight, mg	33.0 ± 1.4^ab^	34.3 ± 2.1^b^	32.4 ± 4.3^a^	32.6 ± 1.6^ab^	33.5 ± 1.5^a^	32.4 ± 1.3^a^	34.1 ± 1.4^b^
Relative aorta weight, %	0.02 ± 0.001^a^	0.03 ± 0.003^bc^	0.03 ± 0.003^b^	0.03 ± 0.003^ab^	0.03 ± 0.003^b^	0.02 ± 0.002^a^	0.03 ± 0.003^c^

Data are presented as mean ± SD (n = 12/group). Values in a row not sharing the same superscript letter are significantly different at *p* < 0.05. HF: high fat diet + cholesterol; post FO: fish oil diet after induction with HF diet; post SP: spirulina diet after induction with HF diet; Post SF: spirulina and fish oil diet after induction with HF diet; Pre-SF: pre-treatment with spirulina and fish oil diet before induction with HF diet; HF + SF: HF diet combined with spirulina and fish oil.

### Effect of treatments on plasma and aortic lipids

Hypercholesterolemia alters the lipid profile of the hamsters. The HF group had significantly higher plasma TC, HDL-C and LDL-C levels, as well as elevated atherogenic index and aortic TC level compared with the control group (*p* < 0.05) (Table 3). Plasma TC level of the hamsters in all groups was significantly different from the control group (*p* < 0.05). The post SP group had the lowest plasma TC levels among the groups induced with hypercholesterolemia for 4 weeks, and the HF + SF group had the highest plasma TC levels compared with the control and HF groups (*p* < 0.05). The addition of fish oil could not lower plasma TC levels and hence the post FO, post SF, and pre-SF groups had similar plasma TC levels. A similar trend was observed with plasma LDL-C levels; the higher plasma TC levels, the greater plasma LDL-C levels and vice versa. The post SF group had the highest plasma TG levels among the pre- and post-treatment groups (*p* < 0.05). Plasma TG levels in the HF + SF group were higher than those in the HF and control groups (*p* < 0.05). Plasma TG levels in the HF, post FO, post SP, and pre-SF groups did not differ significantly from the control group. Plasma HDL-C levels were higher in the HF, post SP, pre-SF, and HF + SF groups compared with those in the control group (*p* < 0.05), and lower in the post FO and post SF compared with those in the post SP and pre-SF groups (*p* < 0.05). The post SP group had the highest plasma HDL-C levels among other post- and pre-treatment groups. The HF + SF group had decreased plasma HDL-C levels compared with the HF group (*p* < 0.05). Plasma HDL-C levels in the post FO and post SF groups were even similar to those in the control group. The ratio of TC to HDL-C was significantly lower in the post SP group, but significantly higher in the post FO group compared with other post- and pre-treatment groups (*p* < 0.05). The HF + SF group had increased the ratio of TC to HDL-C compared with the HF group (*p* < 0.05). The atherogenic index of plasma was significantly higher in the HF group compared with that in the control and HF + SF groups (*p* < 0.05), and was significantly lower in the post SP group compared with that in the control, post FO, post SF, and pre-SF groups (*p* < 0.05). This finding suggests that a high cholesterol diet is more atherogenic, while the spirulina diet (post SP) and pre-SF treatment is less atherogenic. The findings of aortic lipid concentration showed that the HF group had the highest accumulation of TC in the aorta compared with the control and HF + SF groups (*p* < 0.05).

**Table 3:** **Treatment effects on plasma and aortic lipids in the hamsters**

	Control	HF	Post FO	Post SP	Post SF	Pre-SF	HF + SF
TC, mmol/l	7.2 ± 1.3^a^	23.1 ± 6.7^c^	28.7 ± 3.0^d^	15.5 ± 1.4^b^	28.6 ± 4.8^d^	27.1 ± 7.4^cd^	48.1 ± 6.7^e^
TG, mmol/l	5.5 ± 1.6^ab^	6.6 ± 0.9^ab^	7.4 ± 1.2^b^	7.9 ± 2.7^b^	9.7 ± 2.0^c^	7.5 ± 2.2^b^	10.3 ± 2.7^c^
HDL-C, mmol/l	3.6 ± 1.1^a^	7.2 ± 3.1^cd^	3.6 ± 0.6^a^	7.5 ± 1.1^d^	4.3 ± 0.6^ab^	6.1 ± 1.5^c^	5.0 ± 1.1^b^
LDL-C, mmol/l	2.9 ± 1.8^a^	14.5 ± 5.9^b^	23.6 ± 3.0^c^	6.4 ± 1.2^a^	22.4 ± 4.5^c^	19.5 ± 7.3^c^	41.0 ± 6.4^d^
TC/HDL-C	2.1 ± 0.8^a^	5.1 ± 6.1^bc^	8.0 ± 1.6^d^	2.1 ± 0.3^a^	6.8 ± 1.3^c^	4.7 ± 1.8^b^	10.1 ± 2.6^d^
AIP, Log (TG/HDL-C)	0.16 ± 0.05^b^	0.43 ± 0.16^d^	0.30 ± 0.12^c^	0.12 ± 0.06^a^	0.28 ± 0.04^c^	0.14 ± 0.12^b^	0.31 ± 0.15^c^
Aortic TC, nmol/mg aorta	1.0 ± 0.5^a^	9.9 ± 20.9^b^	4.6 ± 4.2^ab^	2.0 ± 1.3^a^	1.3 ± 0.9^a^	2.0 ± 1.7^a^	2.1 ± 1.7^a^

Data are presented as mean ± SD (n = 12/group). Values in a row not sharing the same superscript letter are significantly different at *p* < 0.05. HF: high fat diet + cholesterol; post FO: fish oil diet after induction with HF diet; post SP: spirulina diet after induction with HF diet; Post SF: spirulina and fish oil diet after induction with HF diet; Pre-SF: pre-treatment with spirulina and fish oil diet before induction with HF diet; HF + SF: HF diet combined with spirulina and fish oil. TC: Total cholesterol; TG: Triglycerides; HDL-C: High density lipoprotein-cholesterol; LDL-C: Low-density lipoprotein-cholesterol; AIP: atherogenic index of plasma.

### Effect of treatments on hepatic lipid peroxidation and antioxidant status

**Figure 1:**
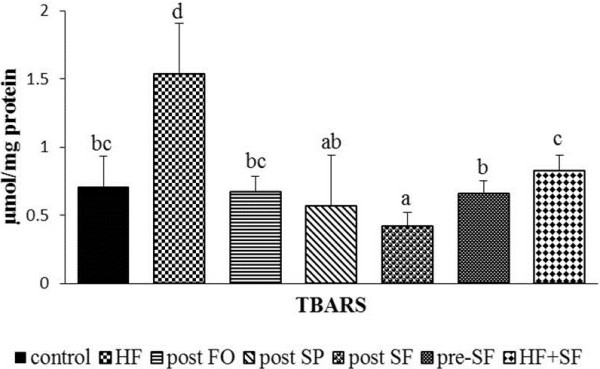
**Effect of fish oil, spirulina, and their combination on lipid peroxidation expressed as thiobarbituric acid reactive substances (TBARS) in liver.** Data are presented as mean ± SD (n = 12/group). Values not sharing the same letter are significantly different at *p* < 0.05. HF: high fat diet + cholesterol; post FO: fish oil diet after induction with HF diet; post SP: spirulina diet after induction with HF diet; Post SF: spirulina and fish oil diet after induction with HF diet; Pre-SF: pre-treatment with spirulina and fish oil diet before induction with HF diet; HF + SF: HF diet combined with spirulina and fish oil.

**Figure 2:**
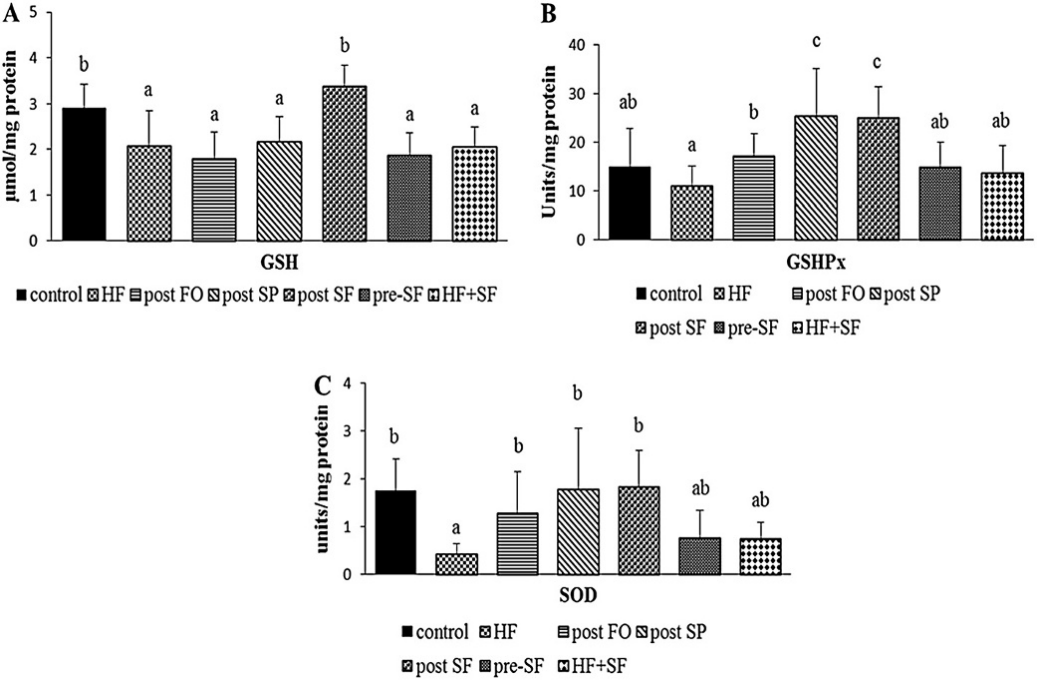
**Effect of fish oil, spirulina, and their combination on (A) GSH concentration, (B) GSHPx, and (C) SOD activities in liver.** Data are presented as mean ± SD (n = 12/group). Values not sharing the same letter are significantly different at *p* < 0.05. HF: high fat diet + cholesterol; post FO: fish oil diet after induction with HF diet; post SP: spirulina diet after induction with HF diet; Post SF: spirulina and fish oil diet after induction with HF diet; Pre-SF: pre-treatment with spirulina and fish oil diet before induction with HF diet; HF + SF: HF diet combined with spirulina and fish oil. GSH: glutathione; GSHPx: glutathione peroxidase; SOD: superoxide dismutase.

We determined the effectiveness of the treatments on protecting against lipid peroxidation and improving the antioxidant defense system in the presence of hypercholesterolemia. A high cholesterol diet increased the level of lipid peroxidation in the liver above control levels (1.5 ± 0.3 vs 0.7 ± 0.2 μmol/mg protein, *p* < 0.05) (Figure 1). While both single and combined treatments of spirulina and fish oil reduced lipid peroxidation level to near or below control levels, the post SF group had the greatest reduction in hepatic lipid peroxides (post FO, 0.7 ± 0.1, pre-SF, 0.7 ± 0.1, and post SF, 0.4 ± 0.1 μmol/mg protein, *p* < 0.05). This finding suggests that single or combined use of spirulina and fish oil may prevent or treat lipid peroxidation. To further understand the protective effect of the treatments in this study, several antioxidant enzymes in the liver were analyzed. Hepatic GSH level was decreased in the HF group compared with that in the control group, but not different from the HF + SF group (Figure 2A). The post SF group had the highest GSH level (3.4 ± 0.5 μmol/mg protein, *p* < 0.05) compared with the post FO, post SP, and pre-SF groups except the control group (Figure 2A). The use of spirulina as a single treatment or in combination with fish oil increased GSHPx activity compared with other post-treatment groups and the control group (post FO, 17.1 ± 4.6, post SP, 25.3 ± 9.7, and post SF, 25.0 ± 6.3 unit/mg protein, *p* < 0.05) (Figure 2B). The activity of SOD was lower in the HF group compared with the control, but did not differ from the HF + SF group (0.4 ± 0.2, 1.7 ± 0.7, and 0.7 ± 0.3 unit/mg protein, respectively, *p* < 0.05). However, hepatic SOD activity in the control, post FO, post SP, post SF, and pre-SF (1.7 ± 0.7, 1.3 ± 0.8, 1.8 ± 1.3, 1.8 ± 0.8, and 0.8 ± 0.6 unit/mg protein) groups did not differ significantly (Figure 2C).

### Effect of treatments on aortic inflammation

Hypercholesterolemia and inflammation are key processes in the pathogenesis of atherosclerosis. In this study, both pro- and anti-inflammatory cytokines in the aorta were investigated. The HF group increased pro-inflammatory cytokines, TNF-α and IL-1β, in the aorta compared with the control group (*p* < 0.05) (Table 4). Aortic TNF-α concentrations were higher in the post FO, post SF, pre-SF, and HF + SF groups compared with those in the control group, and also increased in the pre-SF group compared with those in the post FO, post SP, and post SF groups. While IL-1β concentrations in the aorta were increased in the post SF, pre-SF, and HF + SF groups compared with those in the control group (*p* < 0.05), aortic IL-1β concentrations did not differ significantly in the control, post FO, and post SP groups. On the contrary, the post FO, post SP, post SF, and pre-SF groups elevated aortic level of anti-inflammation cytokine (IL-10) compared with the control group (*p* < 0.05). However, aortic TNF-α, IL-1β, and IL-10 levels were not different between the HF and HF + SF groups.

**Table 4:** **Treatment effects on aortic cytokine concentrations**

	Control	HF	Post FO	Post SP	Post SF	Pre-SF	HF + SF
TNF-α, pg/mg aorta	10.0 ± 1.2^a^	13.8 ± 2.3^c^	11.5 ± 0.7^b^	11.2 ± 0.5^ab^	11.8 ± 1.2^b^	12.8 ± 1.2^c^	12.8 ± 1.5^c^
IL-1β, pg/mg aorta	3.9 ± 0.7^a^	5.7 ± 10.0^c^	4.6 ± 0.5^ab^	4.5 ± 0.4^ab^	4.8 ± 0.5^b^	4.8 ± 0.4^b^	5.2 ± 1.3^bc^
IL-10, pg/mg aorta	7.6 ± 7.0^a^	8.1 ± 3.4^a^	15.6 ± 4.2^bc^	19.4 ± 6.7^c^	16.4 ± 4.4^c^	12.8 ± 3.8^b^	7.4 ± 10.0^a^

Data are presented as mean ± SD (n = 12/group). Values in a row not sharing the same superscript letter are significantly different at *p* < 0.05. HF: high fat diet + cholesterol; post FO: fish oil diet after induction with HF diet; post SP: spirulina diet after induction with HF diet; Post SF: spirulina and fish oil diet after induction with HF diet; Pre-SF: pre-treatment with spirulina and fish oil diet before induction with HF diet; HF + SF: HF diet combined with spirulina and fish oil. TNF-α: tumor necrosis factor- α, IL-1β: interleukin-1 β, IL-10: interleukin-10.

## Discussion

The severity of hypercholesterolemia, the pathogenesis of atherosclerosis, and CVD risk warrant the need to continually investigate cholesterol-lowering therapies [2]. Hypercholesterolemia, characterized by altered plasma lipids and increased oxidative stress and inflammation, requires both cholesterol-lowering and antioxidant therapies, which were beneficial to patients with atherosclerosis by improving endothelium-dependent vasomotion [42].

Since limited studies have observed the effect of fish oil and spirulina, this study determined the effects of fish oil, spirulina, and their combination on hypercholesterolemia. It was hypothesized that there would be differential effects between fish oil and spirulina, but a combination would be more effective.

The HF + SF group had lower body weight at week 8 compared with the control group, but similar body weight compared with the HF group despite that increased plasma cholesterol and TG levels were observed in the HF + SF group. Decreased body weight and weight gain in the HF + SF group could be attributed to lower food intake and food efficiency, while higher levels of plasma cholesterol and TG could be as a result of lipid synthesis and metabolism. Since the HF + SF group was given a high cholesterol diet supplemented with fish oil, it is possible that high levels of plasma lipids could be as a result of de novo (from the liver) and exo synthesis (from the diet). Our results showed that the tendency that the control and pre-SF groups with higher body weight at week 8 had decreased relative aorta weight, which could be because pre-treatment with spirulina and fish oil effectively decreased the increased relative aorta weight caused by a high cholesterol diet to the similar value as the control group. However, it cannot rule out that the variation for aorta wet weight might be affected by a small deviation during the removal of the aorta manually. A high cholesterol diet generally increased all plasma lipids, and was atherogenic because cholesterol was accumulated in the aorta. Elevated plasma lipids have been associated with adverse cardiovascular outcomes. High total cholesterol level has been shown to alter vascular structure and interfere with endothelial function, while TG and LDL-C levels have been directly linked to poor cardiovascular outcomes [2, 4–6].

In hamsters, fish oil-induced hypercholesterolemia and hyperlipidemia has been shown to be dependent on dietary cholesterol supplementation. The previous studies found that the hamsters fed a high cholesterol diet supplemented with fish oil affected plasma TC and LDL-C levels, but not HDL-C particle [16–18]. Similar to the findings of the previous studies, the present study observed that the post FO diet greatly increased plasma TC and LDL-C levels, but did not increase plasma HDL-C levels. Our results also showed that the HF + SF group increased plasma TC, TG, and LDL-C levels but decreased plasma HDL-C levels compared with the HF group. The adverse effect of the HF + SF diet may be primarily attributed to fish oil rather than spirulina. The possible mechanism for increasing plasma LDL-C levels by fish oil in hamsters may be partially due to decreased hepatic LDL-receptor binding which was dependent on the dietary cholesterol content [18]. In addition, supplementation with fish oil was also found to increase TC:HDL-C ratio even though it did not elevate cholesterol accumulation in the aorta. This particular finding contradicts the findings from the previous studies that have shown the anti-atherogenic effect of fish oil supplementation for a longer period of time [12, 13, 43]. However, fish oil treatment for 2 years lowered plasma TG levels, but did not change the diameter of atherosclerotic coronary arteries in the patients with coronary heart disease [44].

On the other hand, a high cholesterol diet with spirulina decreased TC and LDL-C while increasing HDL-C. This observation is in accordance with the previous studies that demonstrated cholesterol-lowering ability of spirulina regardless of the method used to induce hypercholesterolemia and hyperlipidemia [45–47]. Moreover, the spirulina diet was found to be less atherogenic and hence inhibited cholesterol accumulation in the aorta. The results showed that spirulina can protect against the development of atherosclerosis by lowering blood cholesterol levels [46].

A combination of spirulina and fish oil led to differential results that rejected the hypothesis with regard to lowering cholesterol levels. There was an increment in cholesterol levels when spirulina and fish oil were combined as shown in the post SF, pre-SF, and HF + SF groups. These findings, however, could be attributed to the effect of fish oil which did not lower plasma lipids. Never the less, it would be interesting to note that regardless of elevated cholesterol levels, all combinations inhibited cholesterol accumulation in the aorta, and the pre-SF treatment exhibited lower TC:HDL-C ratio and AIP level. The results suggest that pre-treatment with combined spirulina and fish oil can prevent against atherosclerosis by inhibiting cholesterol accumulation in the aorta.

A number of human and animal studies have demonstrated an increase in oxidative stress in the presence of hypercholesterolemia. In these studies, the induction of hypercholesterolemia increased lipid peroxidation in tissues and serum while reducing the antioxidant enzymes such as GSHPx and SOD activities [7–9, 37]. Likewise in this study, hypercholesterolemia increased lipid peroxidation, and decreased hepatic GSH level, GSHPx, and SOD activities in hamsters. Glutathione has been shown to play a major role in the antioxidant defense system and its deficiency increases the levels of oxidative stress, which could lead to the pathogenesis of many diseases including heart related diseases [48]. The protective role of GSHPx and SOD against hydrogen peroxide radicals and superoxide anions, respectively, has been studied. In hypercholesterolemic conditions, both GSHPx and SOD activities were decreased [8, 9, 49, 50]. Therefore, increased antioxidants indicate the protection against oxidative stress.

In our study, both fish oil and spirulina inhibited lipid peroxidation and restored the antioxidant status in hamsters. While there was no significant change in hepatic GSH level in the post FO and post SP groups, there were significant increases in hepatic GSH level, GSHPx and SOD activities in the post SF group. According to the findings of Grotto et al. [22], the effect of fish oil on oxidative stress was not related to its antioxidant ability. Though this study assessed the antioxidant status, the oxidation process was not as a result of hypercholesterolemia. Another study showed that fish oil possessed antioxidant properties, and hypercholesterolemia was not a factor either [19]. The previous findings were consistent with our finding that fish oil increased the activity of antioxidant enzymes when hypercholesterolemia was as a factor [23, 24]. Spirulina also increased the activity of antioxidant enzymes. The previous studies have shown that spirulina had antioxidant properties [35, 37, 51]. These findings suggest that both fish oil and spirulina can prevent oxidative stress and further improve the antioxidant status. In addition, only the post SF group but not the pre-SF and HF + SF groups inhibited lipid peroxidation and restored the antioxidant status to above or to control levels, which indicates that a combination of spirulina and fish oil is beneficial only when factors causing oxidative stress are eliminated.

Inflammation and hypercholesterolemia have been linked to the pathogenesis of cardiovascular disease. Consequently, cytokines have been shown to play an important role in the pathogenesis of cardiovascular disease and hence they become an important target for therapy [52, 53]. While the role of TNF-α and IL-1β in the pathogenesis of atherosclerosis is linked to the recruitment of macrophages and the accumulation of cholesterol in the arterial wall [54, 55], IL-10 works as an anti-inflammatory cytokine which inhibits inflammation and limits adverse outcomes in cardiovascular disease [56]. In our study, a high cholesterol diet increased inflammation by increasing TNF-α and IL-1β and reducing the anti-inflammatory cytokine IL-10. Furthermore, single treatment of fish oil or spirulina regulated inflammation by decreasing the concentration of TNF-α and IL-1β and increasing the concentration of IL-10. However, there were differential results that were observed when spirulina and fish oil were combined. The pre-SF and HF + SF groups increased TNF-α concentration, while the post-SF and pre-SF groups increased anti-inflammatory cytokine IL-10. The previous studies supported that fish oil modulated inflammatory cytokines by inhibiting the activation of transcription factor nuclear factor-κB [57, 58]. Spirulina exhibited its ability in inhibiting inflammation. The anti-inflammatory effect of spirulina may be attributed to c-phycocyanin, a selective inhibitor of cyclooxygenase-2 [59, 60].

The limit of this study is that the experimental diet was not maintained a high cholesterol diet for entire 8 weeks in all the treatment groups, therefore, the comparison between the treatment groups and the HF group was only performed between the HF + SF and HF groups which were given a high cholesterol diet for 8 weeks. Whereas other treatment groups were fed combined high cholesterol and control diets, and each diet was given for 4 weeks, the comparisons were made among the pre- and post-treatment groups rather than the HF group. The study for determining the effects of the treatments in the animals given a high cholesterol diet can be further conducted in the future. In addition, the results of this study can be applied to future human study. Human equivalent doses of fish oil and spirulina translated from hamsters are 1.0 and 0.1 mg/kg body weight, respectively, using the body surface area normalization method and the conversion *K*_m_ factor of 0.135 from 0.08-kg hamsters to 60-kg adults [61].

## Conclusions

Consumption of a high cholesterol diet ad libitum possesses detrimental effects on plasma lipids, oxidative stress, and inflammation. Single treatments of fish oil (post FO) and spirulina (post SP) possess protective effects against oxidative stress and inflammation. This effect was also observed when a combination of fish oil and spirulina (post SF) was administered after inducing hypercholesterolemia, unlike when the combination was given before (pre-SF) or together with a high cholesterol diet (HF + SF). Spirulina (post SP) exhibited decreased TC level, LDL-C level, TC:HDL ratio, and atherogenic index of plasma, and increased HDL-C level compared with fish oil (post FO) and combined fish oil and spirulina (post SF) groups. Our findings suggest that the use of spirulina could decrease high cholesterol levels, while fish oil could worsen the lipid levels. In addition, both fish oil and spirulina and their combination (post SF) may be effective in improving oxidative stress and inflammation in hamsters fed a high cholesterol diet.

## Abbreviations

HF: High fat diet + cholesterol
post FO: Fish oil diet after induction with HF diet
post SP: Spirulina diet after induction with HF diet
Post SF: Spirulina and fish oil diet after induction with HF diet
Pre-SF: Pre-treatment with spirulina and fish oil diet before induction with HF diet
HF + SF: HF diet combined with spirulina and fish oil
TC: Total cholesterol
TG: Triglycerides
HDL-C: High density lipoprotein-cholesterol
LDL-C: Low-density lipoprotein-cholesterol
AIP: Atherogenic index of plasma
TNF-α: Tumor necrosis factor-α
IL-1β: Interleukin-1 β
IL-10: Interleukin-10
TBARS: Thiobarbituric acid reactive substances
GSH: Glutathione
GSHPx: Glutathione peroxidase
SOD: Superoxide dismutase.
